# Effect of Sample Volume Variation and Delay in Analysis on Plasma Glucose Concentration in Sri Lankan Healthy Adults

**DOI:** 10.1155/2021/6061206

**Published:** 2021-01-15

**Authors:** Isuru Anupama Dharmasena, Deepani Siriwardhana, Anoja Priyadarshani Attanayake

**Affiliations:** ^1^Department of Medical Laboratory Science, Faculty of Allied Health Science, University of Ruhuna, Galle 80000, Sri Lanka; ^2^Department of Pathology, Faculty of Medicine, University of Ruhuna, Galle 80000, Sri Lanka; ^3^Department of Biochemistry, Faculty of Medicine, University of Ruhuna, Galle 80000, Sri Lanka

## Abstract

The correct volume of sample and time of storing prior to the analysis are important considerations in the estimation of plasma glucose concentration of patients. The present study was to determine the effect of sample volume variation and time delay in the analysis of plasma glucose results in healthy adults. A total of 30 individuals aged between 20 and 30 years were selected for the study. Blood samples were collected into three fluoride-oxalate collection tubes separately. The results revealed that the sample volume variation from 2.0 mL fluoride-oxalate tube to 1.0 mL and 3.0 mL did not significantly affect the plasma glucose concentration (*p* > 0.05). However, the plasma glucose concentration in the sample significantly decreased upon delaying the analysis. The mean fasting plasma glucose concentration of analysis after one hour of collection and analysis after three hours of collection was not significantly different (*p* > 0.05). The mean fasting plasma glucose concentrations between one hour and five hours timepoints after collection (*p* < 0.001) and between three hours and five hours after collection (*p* = 0.014) were significantly different. In conclusion, overfilling and underfilling (2.0 ± 1.0 mL) of fluoride-oxalate tubes did not affect the plasma glucose results significantly. If the samples are analyzed within three hours of collection, the time dependent change too is not statistically significant.

## 1. Introduction

Diabetes mellitus is a noncommunicable metabolic disease characterized mainly by hyperglycemia. Currently, it has become a common public health problem in the world as well as in Sri Lanka. In 2014, the estimated patients with diabetes were approximately 422 million [1]. International Diabetes Federation has predicted that diabetes will affect 642 million people worldwide in 2040 [1]. According to recent reports published by the World Health Organization, the number of diabetes-related deaths in Sri Lanka in 2016 was 10130 (7% of total deaths) [2]. Therefore, early diagnosis of diabetes mellitus is important to manage its severity and prevent the occurrence of its associated complications.

At present, plasma glucose is one of the most common diagnostic tests in clinical laboratories [3]. Out of the total workload in Chemical Pathology Laboratories, 30%–40% are related to the estimation of glucose concentration [4]. Though glycated hemoglobin and plasma glucose testing are both advocated for the diagnosis of diabetes mellitus, the most frequently used primary laboratory test is still the fasting plasma glucose in Sri Lankan setting. In addition, laboratories receive requests for random plasma glucose, postprandial plasma glucose, and oral glucose tolerance tests. Plasma glucose is analyzed using semiautomated or fully automated analyzers or spectrophotometrically based on the facilities available in a given laboratory. Glucose oxidase-peroxidase and hexokinase are the commonly used enzymatic methods for glucose analysis in these platforms [5–7].

Universally, blood is collected to an anticoagulated tube with an antiglycolytic agent for plasma glucose estimation. The current combinations include sodium fluoride/potassium oxalate, buffered sodium citrate, and a combination of sodium fluoride/potassium oxalate or citrate and EDTA for the estimation of the plasma glucose level [8]. Though glucose can be measured using serum by collecting blood to plain tubes, studies have proven that glucose concentration is higher in plasma than in serum because of the low water content in plasma [3]. To obtain an accurate value in the estimation of plasma glucose result, it is better to use powder form anticoagulant containing tubes to prevent the dilutional effect of blood by a liquid anticoagulant. According to literature, mixing of 100 µL of liquid sodium fluoride-potassium oxalate in 2.7 mL of blood can cause a 3.7% of dilution [9].

However, the most critical point in sample collection is the inhibition of in vitro glycolysis in the collection tube. In Sri Lankan clinical practice, sodium fluoride/potassium oxalate tubes are used as collection tubes for the estimation of glucose concentration. Fluoride binds with inorganic phosphates to form fluorophosphates that bind with magnesium ions in the presence of enolase enzyme [3]. Then, it inhibits the action of enolase and therefore inhibits a late step of glycolysis. As a result, the plasma glucose level is stabilized. Though fluoride is an inhibitor, the action is not immediate to inhibit in vitro glycolysis [10]. When samples are kept for one hour, at room temperature, 5%–7% of glucose is reduced per hour according to the previous observations [11].

According to the amount of anticoagulant, there is a recommended blood volume to be filled into the tube. The ratio of fluoride to blood is 2 mg/mL. Oxalate to blood ratio can be within 1-2 mg/mL. Incorrect sample volume is an important parameter in sample acceptance or rejection criteria [12]. In Sri Lankan hospital setup, underfilling of the tube is more common than overfilling of the tube for the estimation of the plasma glucose level. This is due to problems associated with patients with difficult venipuncture or inadequate drawing of blood if multiple tests have to be performed. If the recommended volume is changed, glycolytic inhibition may not be optimal. Therefore, it is important to keep the correct volume ratio of blood to anticoagulant for the accurate plasma glucose estimation.

In addition, the turnaround time is also an important consideration in plasma glucose estimation. Prolonged contact of plasma with cellular part of blood leads to the reduced plasma glucose level due to utilization by red cells, white cells, and platelets. According to the standard operating procedures adopted locally, plasma glucose estimation should be performed within two hours after the collection of sample, and sample preparation (separation of plasma from cells) for the analysis should be performed within one hour of collection [13]. It is difficult to transport and prepare samples for the analysis within one hour when samples are collected at satellite collection centers. We herein determine the effect of sample volume variation and time delay in the analysis of plasma glucose results in healthy adults.

## 2. Materials and Methods

Sigma 2-5 centrifuge machine (Sigma Laborzentrifugen GmbH, Osterode am Harz, Germany), Vision KMC-1300V vortex mixture (Vision Scientific, Gyeonggi-do, Korea), and Shimadzu UV-1800 spectrophotometer (Shimadzu Corporation, Japan) were used in the sample preparation and in absorbance measurement, respectively.

Commercially available Shandong Chengwu (China) fluoride-oxalate collection tubes (preferred manufacturers' volume −2.0 mL) were used to collect blood from participants.

### 2.1. Study Protocol

Blood was collected from 30 participants (15 healthy males and 15 healthy females) aged between 20 and 30 years, following 8–10 hours of fasting. Any individual on any medication, with difficult venous access or diagnosed with diabetes mellitus, was excluded from the study. All blood samples were collected after obtaining written consent. Blood drawing was performed according to the standard operating procedures by a trained nursing officer.

### 2.2. Ethical Considerations

Ethical approval was granted from the Ethical Review Committee of Faculty of Allied Health Sciences, University of Ruhuna, Sri Lanka (reference number: 14.02.2018:013).

Each participant fasted for 8–10 hours before collection of blood. A 6.5 mL of blood was drawn from each person and dispensed into three fluoride-oxalate collection tubes as 1.0 mL, 2.0 mL, and 3.0 mL separately. Samples were mixed gently. Within 40–50 minutes of collection, samples were centrifuged at 3000 rpm for 5 minutes to obtain plasma, and an aliquot was taken from the tube for glucose analysis at each timepoint. The fasting plasma glucose concentration was estimated after 1 hour, 3 hours, and 5 hours of collection according to the glucose oxidase-peroxidase method using a commercially available spectrophotometric enzyme assay kit (Biorex Diagnostics, UK) [14]. Each day, two levels of in-house prepared quality control samples (normal and high) were run to check the precision of the results.

A volume of ten microliters (10 *µ*L) of plasma samples or standard were mixed with 1.0 mL of glucose reagent and incubated 5 minutes at 37°C. The absorbance of each sample was measured using a spectrophotometer at 500 nm. Plasma glucose concentration was determined.

### 2.3. Statistical Analysis

Data were analyzed using appropriate statistical tests; means were compared by analysis of variance (ANOVA) followed by Tukey HSD used for multiple comparisons. The values of *p* < 0.05 were considered statistically significant. The assumptions were verified for the ANOVA *p* values and Tuckey HSD to be valid (Supplementary material, Figures 1–3). Data analysis was performed using IBM SPSS version 20 software.

## 3. Results

Analysis phases were defined as “1 hour, 1.0 mL; 1 hour, 2.0 mL; 1 hour, 3.0 mL; 3 hours, 1.0 mL; 3 hours, 2.0 mL; 3 hours, 3.0 mL; 5 hours, 1.0 mL; 5 hours, 2.0 mL; and 5 hours, 3.0 mL.” Mean plasma glucose value of all phases was compared with each other at the significance level of 0.05. The mean plasma glucose concentration in different phases in analysis is mentioned in Table 1.

**Figure 1:**
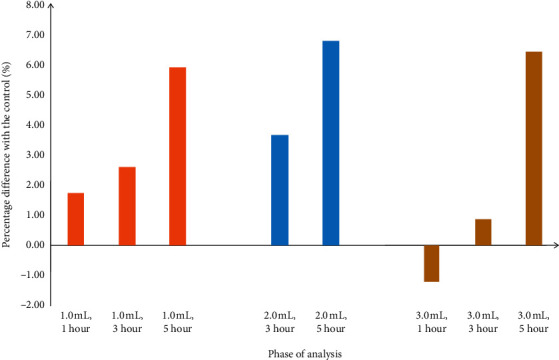
Percentage differences of mean values in each phase of analysis.

A summary of percentage differences of mean values in each phase of analysis is shown in Figure 1.

The results of the present investigation showed that there was no statistically significant difference between different volume groups at the same analysis time (*p* = 0.535) and in the analysis time and sample volume (time*∗*volume) (*p* = 0.818). However, when considering sample analysis time at a fixed volume, there was a statistically significant difference between analyses (*p* < 0.05).

The mean fasting plasma glucose concentration of analysis after one hour of collection and analysis after three hours of collection was not significantly different (*p* > 0.05). The mean fasting plasma glucose concentrations between one hour and five hours timepoints after collection (*p* < 0.001) and between three hours and five hours after collection (*p* < 0.001) were significantly different (Table 2).

## 4. Discussion

The present research aimed to determine the effect of sample volume variation coupled with time delay in the analysis of plasma glucose concentration in healthy adults. Our results showed that sample volume variation alone from 1 mL to 3 mL did not contribute to a statistically significant difference to the measured plasma glucose concentration when blood was collected to fluoride-oxalate tubes. However, when constant sample volumes were considered, there was a statistically significant change in glucose concentration between the timepoints of one and five hours and three and five hours.

Preanalytical factors affecting the reliability of plasma glucose results have been recently reviewed [1]. The type of tube and the time of analysis since blood collection are the key considerations, which are both relevant to the stability of glucose. The debate on the tube with the best additive to minimize glycolysis is still unresolved. Until more evidence-based alternatives are found, Pasqualetti et al. [1] recommend using fluoride-oxalate tubes for collecting blood for plasma glucose. Fluoride-oxalate tube is the most common tube used in the local setting; hence, we evaluated the effects of sample volume and time delay on glucose stability. In our study, the reference sample was the tube filled up to the optimum volume (2 mL) and analyzed at one hour. Hence, the changes in glucose concentration occurring during the first hour after collection were not assessed, which is a limitation in our study. Dimeski et al. [8] showed that there was a 5.4%, 7%, and 8.5% change in glucose concentration at one hour, two hours, and four hours from zero hour. Our study showed 3.5% and 6.7% change at 3 hours and 5 hours after collection compared to the reference sample (2 mL, 1 hour). Our study population consisted of healthy subjects. Apart from being a diagnostic test, fasting plasma glucose is utilized to assess the glycemic control of patients with type 2 diabetes mellitus in the local setting. Hence, it is warranted to evaluate the effect of volume and time delay in plasma glucose assay of patients with diabetes, which has not been assessed in the current study.

The novelty of our study is assessing the effect of volume variation on the stability of glucose. As there was no apparent statistically significant difference between sample volumes of 1–3 mL, the sample rejection practice solely based on volume needs reevaluation to determine whether there is any clinically significant change. Sometimes, there are inadvertent delays in sample transportation even from wards and clinics in a hospital. This is more of a problem when samples are coming from collection centers to a main laboratory. Unnecessary rejection based on sample volume variation will delay diagnosis and inconvenience both the patient and the clinician. Hence, we recommend more judicious application of acceptance rejection criteria related to sample volume in plasma glucose analysis.

Most of the research studies have proven that sodium fluoride is an ineffective antiglycolytic agent up to 2–4 hours of sample collection [8, 10, 11]. However, it is practically difficult to collect, transport, and analyze samples within one hour. The following recommendations could be suggested to minimize the effect of plasma glucose estimation by delayed transportation, namely, collection of samples at the laboratory, separation of plasma just after the collection from cellular part, and transport in cold containers until more robust evidence is found regarding other antiglycolytic agents such as citrate. In facilities where it is impractical to separate plasma from cells, soon after collection, the plasma glucose analysis if performed at least within three hours of collection, there will be less variation in plasma glucose results based on our study findings.

## 5. Conclusion

Overfilling and underfilling (2.0 ± 1.0 mL) of fluoride-oxalate tubes did not affect the plasma glucose results significantly. If the samples are analyzed within three hours of collection, the time-dependent change too is not statistically significant.

## Figures and Tables

**Table 1:** Mean plasma glucose concentration in different phases in analysis.

Duration between the collection and analysis (hours)	Sample volume (mL)	Mean plasma glucose concentration (mmol/L)
1	1.0	5.69 ± 0.10
3	1.0	5.64 ± 0.10
5	1.0	5.45 ± 0.11
1	2.0	5.79 ± 0.11
3	2.0	5.58 ± 0.10
5	2.0	5.40 ± 0.09
1	3.0	5.86 ± 0.09
3	3.0	5.74 ± 0.10
5	3.0	5.42 ± 0.09

The values are expressed as mean ± SD.

**Table 2:** Mean comparison of different analysis times at constant sample volumes.

Time between sample collection and analysis (hours)	Mean fasting plasma glucose concentration (mmol/L)
1	5.78^a^ ± 0.06
3	5.65^a^ ± 0.06
5	5.42^b^ ± 0.06

The values are expressed as mean ± SD. ^a^No statistically significant difference in mean fasting plasma glucose concentration between analysis after one hour of collection and analysis after three hours of collection (*p* > 0.05). ^b^A statistically significant difference in mean fasting plasma glucose concentration between analysis after one hour of collection and analysis after five hours of collection (*p* < 0.001) and between the analysis after three hours and five hours after collection (*p* < 0.001).

## Data Availability

The mean values to support the findings of this study are included in the article, and the raw data are available from the corresponding author upon request.

## Supplementary Materials

Supplementary Materials The assumptions were verified for the ANOVA *p* values and Tuckey HSD to be valid. Figure 1. Scatter plot of residuals for plasma glucose concentration vs. the predicted value for glucose concentration. No any specific pattern in the scatter plot. The variances are equal at any level (assumption 1). The significance of all the above heteroskedasticity tests is *p* > 0.05. Therefore, for any variable (analysis time, sample volume, and sample volume*∗*analysis time), variances are equal at any level. Figure 2. Q-Q plot of residual for plasma glucose concentration. Figure 3. Detrended normal Q-Q plot of residual for plasma glucose concentration. Based on Figure 2 and Figure 3, residuals are normally distributed (assumption 2).
