# Supplementary material for: Effect of Sample Volume Variation and Delay in Analysis on Plasma Glucose Concentration in Sri Lankan Healthy Adults
Source: Scientifica (Cairo). 2021 Jan 15;2021:6061206. doi: 10.1155/2021/6061206 (PMC7822684; doi:10.1155/2021/6061206)
Supplement: Supplementary Materials — The assumptions were verified for the ANOVA p values and Tuckey HSD to be valid. Figure 1. Scatter plot of residuals for plasma glucose concentration vs. the predicted value for glucose concentration. No any specific pattern in the scatter plot. The variances are equal at any level (assumption 1). The significance of all the above heteroskedasticity tests is p > 0.05. Therefore, for any variable (analysis time, sample volume, and sample volume∗analysis time), variances are equal at any level. Figure 2. Q-Q plot of residual for plasma glucose concentration. Figure 3. Detrended normal Q-Q plot of residual for plasma glucose concentration. Based on Figure 2 and Figure 3, residuals are normally distributed (assumption 2). [file 6061206.f1.docx]

**Supplementary material**

**Effect of Sample Volume Variation and Delay in Analysis on Plasma Glucose Concentration in Sri Lankan Healthy Adults**

Isuru Anupama Dharmasena,^1^ Deepani Siriwardhana,^2^ Anoja Priyadarshani Attanayake ^3*^

^1^Department of Medical Laboratory Sciences, Faculty of Allied Health Sciences, University of

Ruhuna, Galle 80000, Sri Lanka.

^2^Department of Pathology, Faculty of Medicine, University of Ruhuna, Galle 80000, Sri Lanka.

^3^Department of Biochemistry, Faculty of Medicine, University of Ruhuna, Galle 80000, Sri Lanka.

The assumptions were verified for the ANOVA p- values and Tuckey HSD to be valid.

Assumption 1: The variable (analysis time, sample volume, sample volume* analysis time) variances are equal at any level.

Figure 1. Scatter plot of residuals for plasma glucose concentration vs predicted value for glucose concentration.

No any specific pattern in the scatter plot. The variances are equal at any level.

The heteroskedastisity test was performed to further confirm the said assumption.

| White test for heteroskedasticity | | |
| --- | --- | --- |
| Chi-Square | df | Significance |
| 1.754 | 8 | 0.988 |

Dependent variable -Fasting plasma glucose concentration

Breusch-Pagan test for heteroskedasticity

| Chi-Square | df | Significance |
| --- | --- | --- |
| 0.038 | 1 | 0.844 |

Dependent variable -Fasting plasma glucose concentration

Modified Breusch-Pagan test for heteroskedasticity

| Chi-Square | df | Significance |
| --- | --- | --- |
| 0.035 | 1 | 0.851 |
| Dependent variable -Fasting plasma glucose concentration | | |

F test for heteroskedasticity

| F | df1 | df2 | Significance |
| --- | --- | --- | --- |
| 0.035 | 1 | 268 | 0.852 |
| Dependent variable -Fasting plasma glucose concentration | | | |

The significance of all the above heteroskedasticity tests is p > 0.05. Therefore, for any variable (analysis time, sample volume, sample volume* analysis time) variances are equal at any level.

Assumption 2: Residuals are normally distributed.

Figure 2. Q-Q plot of residual for plasma glucose concentration

Figure 3. Detrended normal Q-Q plot of residual for plasma glucose concentration

Based on the Figure 2 and Figure 3, residuals are normally distributed.
